# LINC01420 as a oncogenic lncRNA: Expression signatures, functional roles, and prognostic significance in cholangiocarcinoma

**DOI:** 10.1007/s11845-026-04319-w

**Published:** 2026-05-01

**Authors:** Ning Tang, Rongyao Zhou, Rui Sun, Hucheng Ma, Gang Li

**Affiliations:** 1 Division of Hepatobiliary and Transplantation Surgery, Department of General Surgery, Nanjing Drum Tower Hospital, Affiliated Hospital of Medical School, Nanjing University, No. 321, Zhongshan Road, Gulou District, Nanjing, 210008 Jiangsu China; 2 Division of Trauma and Acute Care Surgery, Department of Emergency Medicine, Jinling Hospital, Affiliated Hospital of Medical School, Jinling Hospital, Affiliated Hospital of Medical School, Nanjing University, Nanjing, 210008 Jiangsu China; 3 Pancreatic and Metabolic Surgery, Department of General Surgery, Nanjing Drum Tower Hospital,Affiliated Hospital of Medical School,Nanjing University, No. 321, Zhongshan Road, Gulou District, Nanjing, 210008 Jiangsu China

**Keywords:** Cholangiocarcinoma, LINC01420, miR-320a, ceRNAs, Prognosis, Proliferation

## Abstract

**Background:**

Cholangiocarcinoma (CCA) is a commonly occurred malignancy, and has a worrying survival rate. This study aimed to investigate the relationship between long non-coding RNA LINC01420 and CCA overall survival, and explore the biological function and mechanisms of LINC01420 in CCA progression.

**Methods:**

LINC01420 and miR-320a levels were measured by quantitative real-time PCR. Kaplan-Meier survival curve was performed to analyze the correlation between LINC01420 expression and overall survival. CCK-8 and Transwell assay evaluated CCA cell proliferation, migration, and invasion, respectively. The interaction between LINC01420 and miR-320a by dual-luciferase reporter assay. Bioinformatics analysis predicted miR-320a target genes, related functions and pathway information.

**Results:**

LINC01420 expression was significantly increased in CCA tumor tissues and cells. LINC01420 overexpression was significantly correlated with larger tumor size, lymph node metastasis and advanced TNM stage. LINC01420 was predicted worse overall survival in CCA patients. Knockdown of LINC01420 inhibited CCA cell proliferation, migration and invasion, and these effects could be abolished by the reduction of miR-320a, which was determined as a target of LINC01420 in CCA.

**Conclusion:**

LINC01420 downregulation inhibited tumor progression by acting as a ceRNA of miR-320a in CCA cells. The LINC01420/miR-320a axis may provide novel biomarkers and targets for CCA therapy.

**Supplementary Information:**

The online version contains supplementary material available at https://doi.org/10.1007/s11845-026-04319-w.

## Introduction

Cholangiocarcinoma (CCA) is a heterogeneous malignant tumor that originates from the epithelial cells of the bile duct [1]. The intrahepatic, hilar, and distal bile ducts are among the locations where it occurs [2]. Due to its strong invasiveness and high propensity for lymph node metastasis, as well as low sensitivity to adjuvant therapies like radiotherapy and chemotherapy, patients exhibit poor overall treatment efficacy, significantly shortened survival time, and generally poor prognosis [3, 4]. Therefore, it is urgently needed to find novel strategies to develop CCA prognosis and treatment.

At present, an increasing number of studies have found that long noncoding RNAs (lncRNAs) play major roles in tumor development [5–7]. For instance, MALAT1, a 7.5-kb long lncRNA, was found to have aberrant expression in multiple cancers and is a critical regulator of the metastatic phenotype of lung cancer cells [8, 9]. Lujambio et al. revealed that the aberrant expression of lncRNA T-UCR promotes the progression of colorectal carcinoma [10]. Several researches also revealed that high expression of HOTAIR might be a potential biomarker for Breast Cancer [11, 12]. The differential expression of lncRNAs is closely related to the prognosis of patients and may serve as potential prognostic biomarkers for indicating patient survival prediction [5, 13]. Among them, lncRNA LINC01420 has been confirmed to be highly expressed in both thyroid cancer and nasopharyngeal carcinoma, and is significantly associated with poor prognosis of the patients. Luo et al. suggest that LINC01420 overexpression showed potential as a prognostic marker and potential therapeutic target for TC [14]. Yang et al. found that NPC patients with high LINC01420 expression showed unfavorable prognosis and high LINC01420 modulated cell migration and invasion in NPC [15]. However, whether LINC01420 is associated with disease prognosis in CCA and its biological function in CCA, all remain unknown.

Recently, lncRNAs exert their regulatory function on the expression of key tumor genes by competitively binding with microRNAs (miRNAs) as competitive endogenous RNAs (ceRNAs) [16, 17]. For example, Lian et al. have confirmed that lncRNA AFAP1-AS1 binds to miR-423-5p and activates the Rho/Rac pathway to promote metastasis in nasopharyngeal carcinoma [18]. Cui et al. found that LINC01420 and miR-149-5p form a targeted binding relationship, which promotes the proliferation, migration and invasion of gastric cancer cells [19]. This suggests that LINC01420 may exert a carcinogenic effect in tumors through the ceRNA mechanism. Based on the results of bioinformatics prediction, we further discovered that LINC01420 has potential target binding sites with miR-320a. Given that the functions and regulatory mechanisms of LINC01420 in CCA remain unclear, this study aims to confirm the biological role of LINC01420 in the progression of CCA, and based on the ceRNA theory, deeply explore its interaction with miR-320a. The analysis results of this study may allow researchers to gain new understanding of the function and mechanisms of LINC01420, and provide novel biomarkers and targets for the prognosis and therapy of CCA.

## Materials and methods

### Patients and collection of tissues

The protocols for tissue collection and analysis were all in accordance with the guideline of the Ethics Committee, and have been approved by the Ethics Committee. A signed informed consent was obtained from the patients or guardians before sampling. In this study, tumor tissue samples and adjacent non-tumor tissue samples were collected from 96 CCA patients who underwent curative resection between 2019 and 2023 at Nanjing Drum Tower Hospital, The Affiliated Hospital of Nanjing University Medical School. All tissue samples were verified via histopathology and frozen in liquid nitrogen for further use. Patients who had received any anti-tumor treatment were excluded from this study. Follow-up surveys were conducted by telephone communication or outpatient visits, and survival information of all patients was recorded for further survival analysis.

### Cell culture and transfection

2 CCA cell lines (TFK-1 and HUCCT1) and 1 normal bile duct epithelial cell line (H69) were purchased from the Type Culture Collection of the Chinese Academy of Sciences (Shanghai, China). All cells were cultured in RPMI-1640 medium (Sigma-Aldrich, St. Louis, MO, USA) supplemented with 10% fetal bovine serum (FBS, Invitrogen), 100 U/mL penicillin and 0.1 mg/mL streptomycin, and maintained at 37℃ in a saturated humidified atmosphere with 5% CO_2_.

Mimic negative control (mimic NC), inhibitor NC, small interfering RNA NC (siRNA NC), si-LINC01420, miR-320a mimic and miR-320a inhibitor were synthesized by GenePharma (Shanghai, China). The sequences of the vectors were as follows: si-LINC01420: 5’-CAUCUCAGGUCUCUUGGCUUUGCCA-3’; mimic NC: 5’-UUCUCCGAACGUGUCACGU-3’; miR-320a mimic: 5’-AAAAGCUGGGUUGAG-3’; inhibitor NC: 5’-CAGUACUUUUGUGUAGUACAA-3’; miR-320a inhibitor: 5’-CUCAACCCAGCUUUU-3’. Mimic NC, inhibitor NC, siRNA NC, si-LINC01420, miR-320a mimic, miR-320a inhibitor were transfected into CCA cell lines TFK-1 and HUCCT1 using Lipofectamine 3000 (Invitrogen, CA, USA) following the manufacturer’s protocols. At 48 h post-transfection, evaluating transfection efficiency.

### RNA extraction and quantitative real-time PCR (qRT-PCR)

TRIzol reagent (Invitrogen, Carlsbad, CA, USA) was used to obtain total RNA from tissue samples and cells. Following the manufacturer’s guidelines, the obtained RNA was reversed transcribed into single-stranded cDNA with PrimeScript reverse transcriptase kit (TaKaRa, Shiga, Japan). The expression of LINC01420 and miR-320a was determined using the SYBR green I Master Mix kit (Invitrogen, Carlsbad, California, USA) on a 7500 real-time polymerase chain reaction system (Applied Biosystems, USA). GAPDH and U6 were respectively used to as endogenous control for LINC01420 and miR-320a. Relative expression data were calculated using the 2^−ΔΔCt^ method.

### Cell counting kit (CCK)-8 assay

The proliferation abilities of TFK-1 and HUCCT1 cell lines was evaluated using CCK-8 assay. The cells were seeded into 96-well plate with a density of 1 × 10^3^ cells/well, incubated in a humidified incubator at 37℃ with 5% CO_2_. Until 0, 24, 48, and 72 h, 10 µL CCK-8 reagent was added to the wells respectively, the OD (optical density) at 450 nm was read and recorded.

### Transwell assay

The migration and invasion abilities of TFK-1 and HUCCT1 cell lines were evaluated using Transwell assay. Cell invasion assay was used the Transwell chambers (Corning, NY, USA) precoated with Matrigel (Corning, NY, USA), while cell migration assay was used the Transwell chambers without Matrigel coating. The upper chambers were filled with a serum-free medium, the low chambers were filled with culture medium supplemented with 10% FBS as chemoattractant. The transfected cells were seeded into the upper chamber with a density of 2 × 10^4^ cell/well. After 48 h of incubation, the cells in the lower chambers were stained and observed for counting.

### Dual-luciferase reporter assay

LncBase Predicted v.2 (http://carolina.imis.athena-innovation.gr/diana_tools/web/index.php?r=lncbasev2%2Findex) [20] was used to predict the binding sites of LINC01420 and miR-320a. The mutant (MUT) LINC01420 sequence and the wild type (WT) LINC01420 sequence containing the miR-320a -binding sequence were cloned into the pGL3 (Promega, Madison, WI, USA). LINC01420-MUT and LINC01420-WT along with miR-320a mimic or mimic NC respectively, co-transfected into TFK-1 cells using Lipofectamine 3000 (Invitrogen, CA, USA). After 48 h, the relative luciferase activity was used to measure using dual-luciferase reporter assay system (Promega).

### Bioinformatics analysis

The target genes of miR-320a were predicted through TargetScan, ENCORI and miRDB target gene databases. Online tool STRING was applied to construct PPI network maps of overlapping target genes. The Cytoscape software was used for visualizing the network. Gene function annotation and pathway enrichment analysis of overlapping target genes were conducted through GO and KEGG bioinformatics databases.

### Statistical analysis

SPSS 22.0 (IBM Corp.) and GraphPad Prism 7.0 software (GraphPad Software, Inc.) were used to perform the date statistical analysis. All the measurement data were verified for normal distribution through the Shapiro-Wilk test. Data that follow a normal distribution are analyzed statistically using Student’s t-test (for two groups) or one-way analysis of variance (ANOVA) combined with Tukey’s test (for multiple groups). The relationship between LINC01420 expression and clinicopathological data of CCA patients was examined using Chi-square test. Kaplan-Meier curves were used to establish survival curves of CCA patients, and log-rank test was used to compare the differences between survival curves of CCA patients. Pearson correlation coefficient was applied to confirm the relationship between LINC01420 and miR-320a in CCA patients. Each experiment was repeated at least 3 times. *P* < 0.05 was considered to indicate a statistically significant difference.

## Results

### Overexpression of LINC01420 predicts poor overall survival in CCA patients

**Fig. 1:**
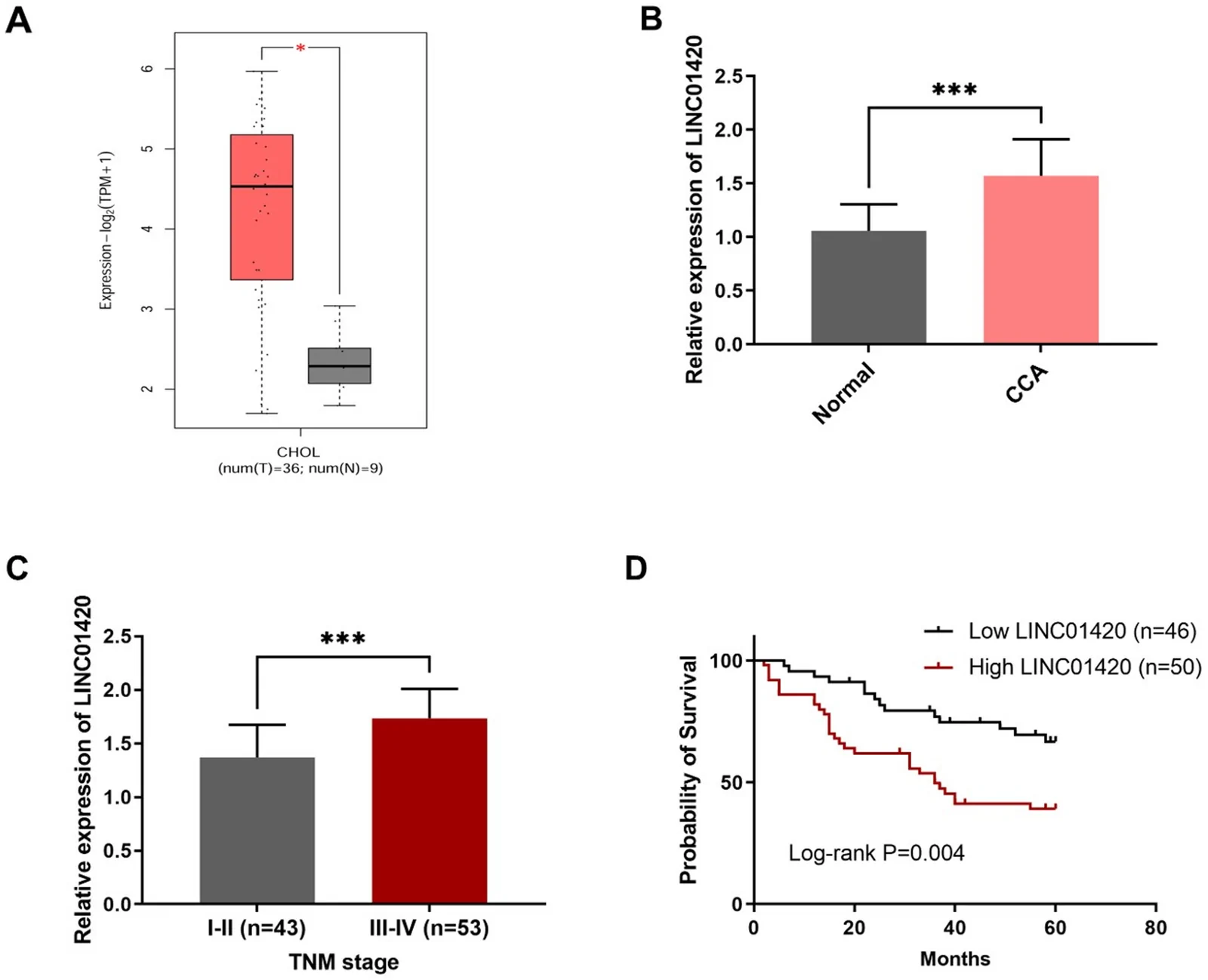
Overexpression of LINC01420 predicts poor overall survival in CCA patients. **A**. Search for LINC01420 being upregulated in CCA using the GEPIA database. **B**. The expression levels of LINC01420 were significantly increased in CCA patients compared with normal. **C**. LINC01420 expression was significantly higher in advanced TNM stage (III-IV) patients than in early TNM stage (I-II) patients. **D**. Kaplan-Meier curves indicated that high LINC01420 was associated with worse overall survival prognosis (Log-rank *P* = 0.004). (** *P* < 0.01, *** *P* < 0.001)

On the public platform GEPIA (http://gepia.cancer-pku.cn/index.html), based on the data from the TCGA database, 36 cases of CCA tissues and 9 cases of normal tissues were extracted for analysis. The results showed that the expression of LINC01420 in CCA tissues was significantly higher than that in normal tissues (Fig. 1A, *P* < 0.05). qRT-PCR was used to measure the expression levels of LINC01420 in CCA tissues and cell lines. Data analysis of tissues from 96 CCA patients included in this study showed that the expression levels of LINC01420 were significantly increased in CCA patients compared with normal (Fig. 1B, *P* < 0.001), and LINC01420 expression was significantly higher in advanced tumor node metastasis (TNM) stage (III-IV) patients than in early TNM stage (I-II) patients (Fig. 1C, *P* < 0.001). Kaplan-Meier curves indicated that high LINC01420 was associated with worse overall survival prognosis (Fig. 1D, Log-rank *P* = 0.004).

### Association of LINC01420 with CCA patients’ clinicopathological features

As shown in Table 1, LINC01420 expression was significantly correlated with tumor size (*P* = 0.021), lymph node metastasis (*P* = 0.004) and TNM stage (*P* = 0.001). Nevertheless, there were no significant associations between LINC01420 expression and age, sex and smoking (all *P* > 0.05). Analysis results of association between LINC01420 expression and clinicopathological features of CCA patients suggested that LINC01420 might be involved in the development of CCA.

**Table 1:** Association of LINC01420 with CCA patients’ clinicopathological features

Variables	No. *n* = 96	LINC01420 expression		*P* value
		Low (*n* = 46)	High (*n* = 50)	
Age (years)				0.762
≤ 60	34	17	17	
> 60	62	29	33	
Sex				0.949
Female	31	15	16	
Male	65	31	34	
Smoking				0.744
Never	35	16	19	
Ever	61	30	31	
Tumor size (cm)				0.021
≤ 3	53	31	22	
> 3	43	15	28	
Lymph node metastasis				0.004
Negative	48	30	18	
Positive	48	16	32	
TNM stage				0.001
I - II	43	29	14	
III - IV	53	17	36	

TNM, Tumor Node Metastasis

### LINC01420 is an independent predictor of CCA prognosis

We conducted univariate and multivariate Cox regression analyses to better understand the prognostic significance of LINC01420 in CCA patients (Table 2). The univariate analysis results showed that tumor size (HR = 0.231, 95% CI: 0.122–0.439, *P* < 0.001), lymph node metastasis (HR = 0.261, 95% CI: 0.134–0.509, *P* < 0.001), TNM stage (HR = 0.292, 95% CI: 0.147–0.579, *P* < 0.001), and LINC01420 expression (HR = 0.072, 95% CI: 0.028–0.185, *P* < 0.001) were all significantly associated with overall survival of patients. The multivariate Cox regression analysis further confirmed that tumor size (HR = 0.443, 95% CI: 0.228–0.863, *P* = 0.017), lymph node metastasis (HR = 0.362, 95% CI: 0.182–0.719, *P* = 0.004), TNM stage (HR = 0.404, 95% CI: 0.199–0.819, *P* = 0.012), and LINC01420 expression (HR = 0.110, 95% CI: 0.041–0.294, *P* < 0.001) are independent prognostic factors for CCA patients.

**Table 2:** Cox regression analysis results

Variables	Univariate analysis			Multivariate analysis		
	HR	95% CI	*P*	HR	95% CI	*P*
Age	1.020	0.552–1.886	0.949	–	–	–
Sex	0.860	0.450–1.645	0.649	–	–	–
Smoking	1.276	0.700-2.329	0.426	–	–	–
Tumor size	0.231	0.122–0.439	< 0.001	0.443	0.228–0.863	0.017
Lymph node metastasis	0.261	0.134–0.509	< 0.001	0.362	0.182–0.719	0.004
TNM stage	0.292	0.147–0.579	< 0.001	0.404	0.199–0.819	0.012
LNC01420	0.072	0.028–0.185	< 0.001	0.110	0.041–0.294	< 0.001

### CCA cell lines have higher LINC01420 expression than normal cells

**Fig. 2:**
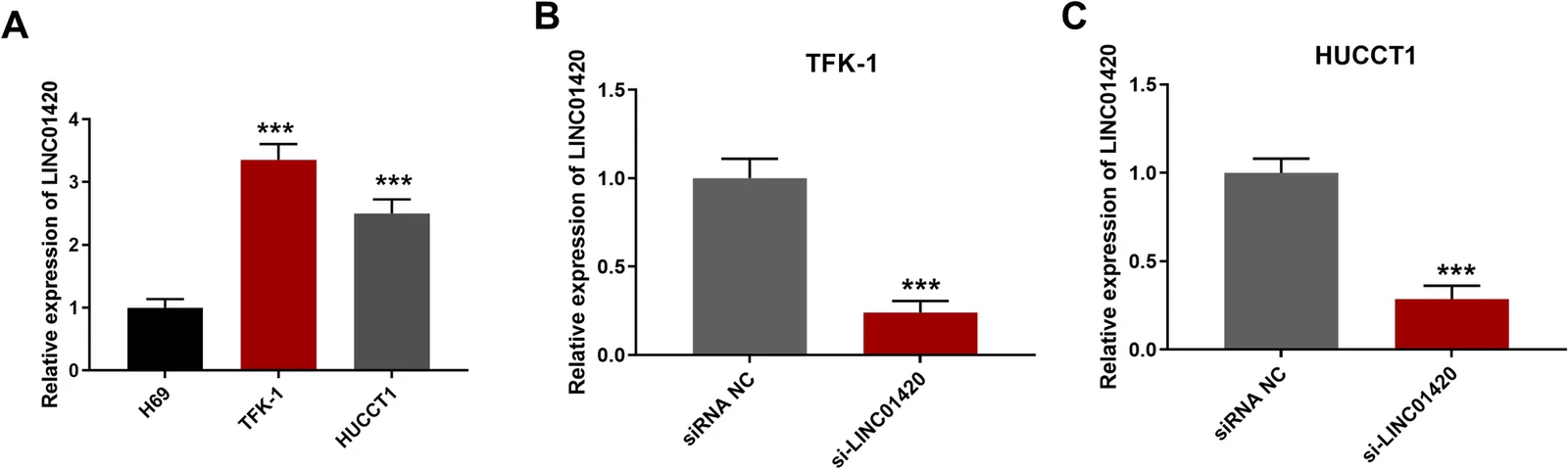
CCA cell lines have higher LINC01420 expression than normal cells. **A**. the expression of LINC01420 was significantly higher in the 2 CCA cell lines (TFK-1 and HUCCT1) compared with normal H69 cell lines. **B**. LINC01420 expressions were significantly reduced after transfection with si-LINC01420 compared with transfection with siRNA NC in TFK-1 cell lines. **C**. LINC01420 expressions were significantly reduced after transfection with si-LINC01420 compared with transfection with siRNA NC in HUCCT1 cell lines. (*** *P* < 0.001)

As shown in Fig. 2A, the expression of LINC01420 was significantly higher in the 2 CCA cell lines TFK-1 (*P* < 0.001) and HUCCT1 (*P* = 0.0003) compared with normal H69 cell lines (all *P* < 0.001). The results of respectively transfection showed that LINC01420 expressions were significantly reduced after transfection with si-LINC01420 compared with transfection with siRNA NC in TFK-1 (Fig. 2B, *P* < 0.001) and HUCCT1 cell lines (Fig. 2C, *P* < 0.001), suggesting that si-LINC01420 significantly inhibited LINC01420 expression.

### Knockdown of LINC01420 inhibits CCA cell proliferation, migration and invasion

**Fig. 3:**
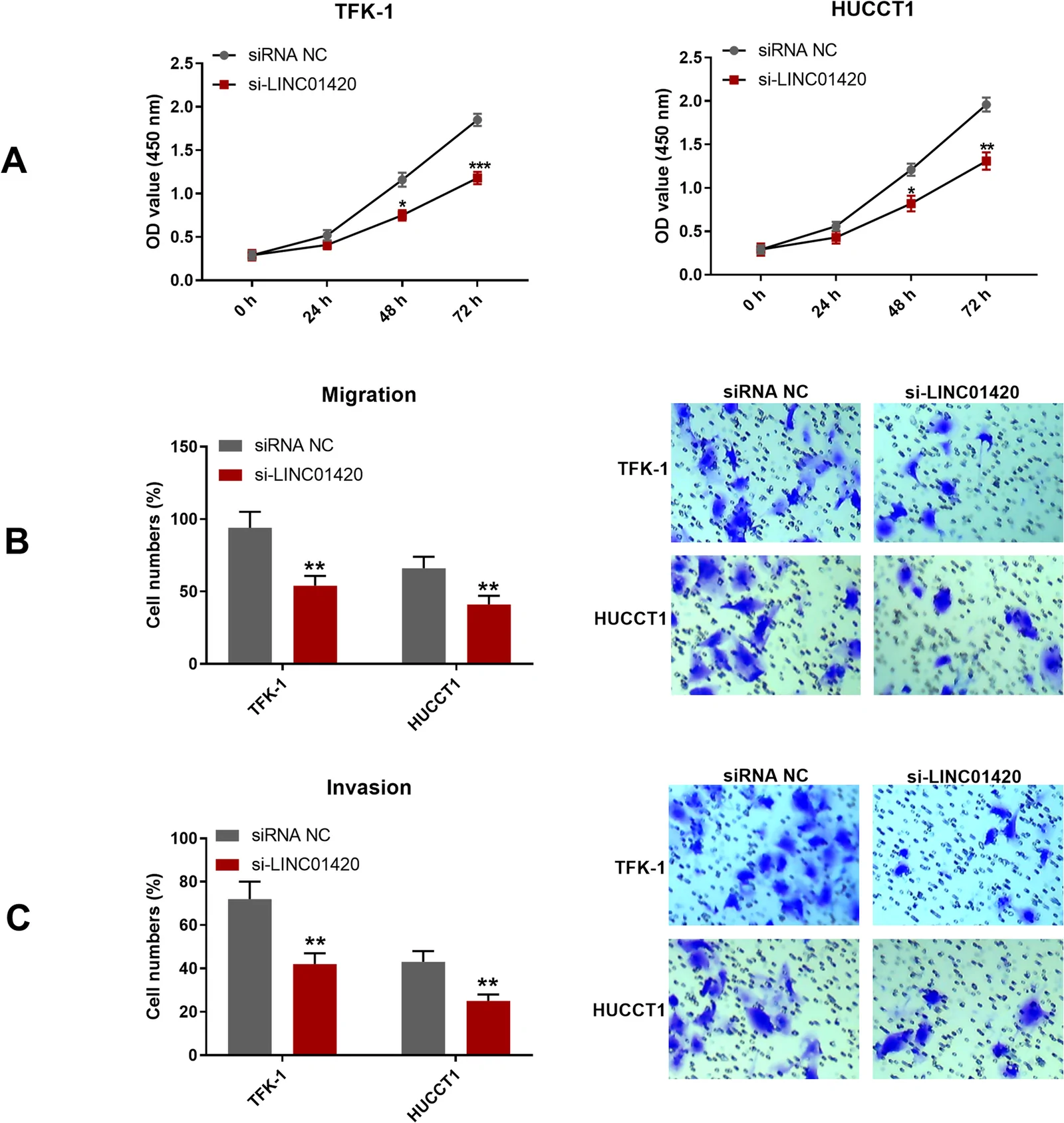
Knockdown of LINC01420 inhibits CCA cell proliferation, migration and invasion. **A**. si-LINC01420 dramatically inhibited the proliferation abilities of TFK-1 and HUCCT1 cell lines compared with siRNA NC. **B**. The migration abilities were observably inhibited by si-LINC01420 compared with siRNA NC. **C**. The invasion abilities were observably inhibited by si-LINC01420 compared with siRNA NC (* *P* < 0.05, ** *P* < 0.01, *** *P* < 0.001)

Cell proliferation assay showed that si-LINC01420 dramatically inhibited the proliferation abilities of TFK-1 (48 h: *P* < 0.05, 72 h: *P* < 0.001) and HUCCT1 cell lines (72: *P* < 0.01) compared with siRNA NC (Fig. 3A). In both TFK-1 cells and HUCCT1 cell lines, the migration abilities (Fig. 3B, all *P* < 0.01) and invasion abilities (Fig. 3C, all *P* < 0.01) were all observably inhibited by si-LINC01420 compared with siRNA NC. Thus, knockdown of LINC01420 inhibited CCA cell proliferation, migration and invasion.

### LINC01420 acts as a ceRNA of miR-320a

**Fig. 4:**
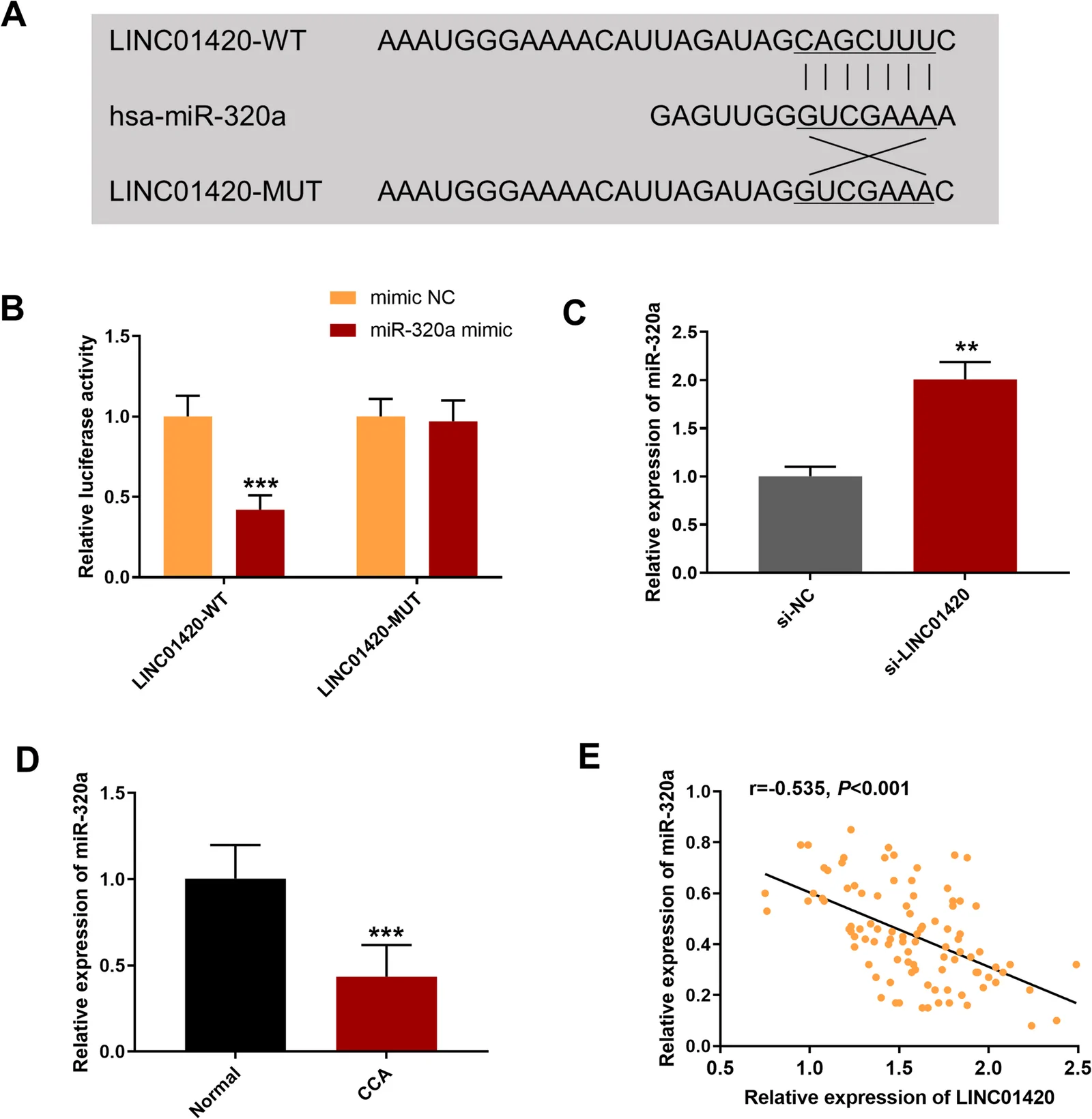
LINC01420 acts as a ceRNA of miR-320a. **A**. The complementary binding sequences and binding sites between LINC01420 and miR-320a. **B**. overexpression of miR-320a inhibited the relative luciferase activity of LINC01420-WT group, whereas no changes in luciferase activity of LINC01420-MUT group. **C**. knockdown of LINC01420 was significantly promoted miR-320a expression in TFK-1 cells. **D**. The expression level of miR-320a was downregulated in CCA tumor tissues compared with the adjacent normal tissues. **E**. miR-320a expression was negatively correlated with LINC01420 expression. (** *P* < 0.01, *** *P* < 0.001)

LncBase Predicted v.2 prediction results showed that LINC01420 and miR-320a were complementary binding sequences, and the complementary binding sequences and binding sites between LINC01420 and miR-320a were shown in Fig. 4A. The results of dual-luciferase reporter assay indicated a direct association between LINC01420 and miR-320a in CCA cells. As presented in Fig. 4B, overexpression of miR-320a inhibited the relative luciferase activity of LINC01420-WT group, whereas no changes in luciferase activity of LINC01420-MUT group (*P* > 0.05). Moreover, knockdown of LINC01420 was significantly promoted miR-320a expression in TFK-1 cells, which suggested that LINC01420 functioned a negative regulatory effect on miR-320a expression (Fig. 4C, *P* = 0.001). As shown in Fig. 4D, the expression level of miR-320a was downregulated in CCA tumor tissues compared with the adjacent normal tissues (*P* < 0.001). And miR-320a expression was negatively correlated with LINC01420 expression as shown in Fig. 4E (*r* = − 0.535, *P* < 0.001). Taken together, we speculated that LINC01420 might act as a ceRNA of miR-320a.

### miR-320a inhibition abolished the changes in TFK-1 cell biological function induced by LINC01420 knockdown

**Fig. 5:**
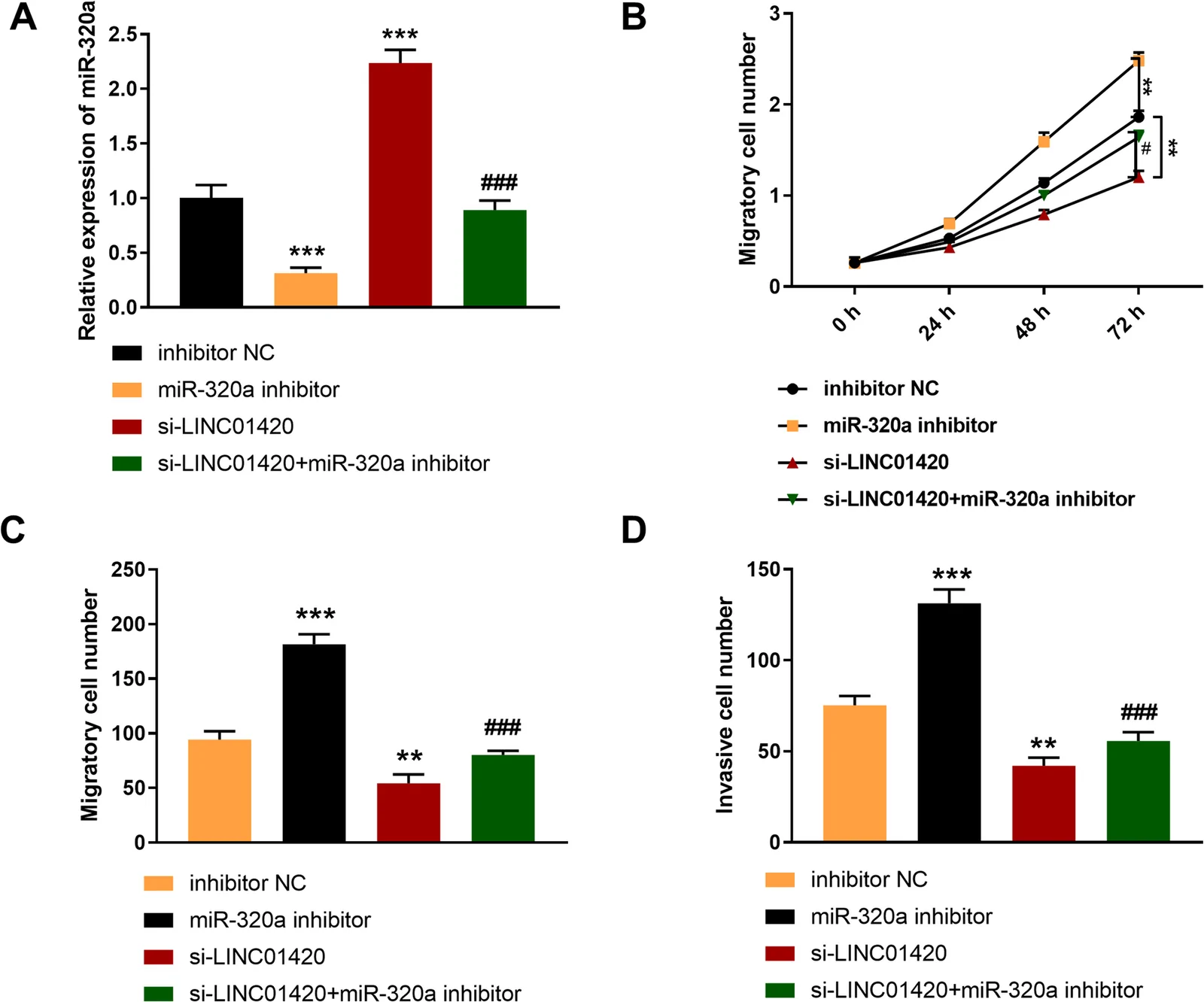
miR-320a inhibition abolished the changes in TFK-1 cell biological function induced by LINC01420 knockdown. **A**. miR-320a inhibitor significantly suppressed miR-320a expression compared with inhibitor NC. LINC01420 knockdown and inhibitor NC promoted miR-320a expression. Co-transfected si-LINC01420 and miR-320a inhibitor indicated that miR-320a inhibitor suppressed the situation in which LINC01420 knockdown promoted miR-320a expression, and decreased the expression level of miR-320a. **B**. miR-320a inhibitor were significantly enhanced the abilities of cell proliferation compared with inhibitor NC. Cell proliferation abilities, which were inhibited by LINC01420 knockdown, were reversed upon miR-320a inhibition and cell functions were instead promoted. **C**. miR-320a inhibitor were significantly enhanced the abilities of cell migration compared with inhibitor NC. Cell migration abilities, which were inhibited by LINC01420 knockdown, were reversed upon miR-320a inhibition and cell functions were instead promoted. **D**. miR-320a inhibitor were significantly enhanced the abilities of cell invasion compared with inhibitor NC. Cell invasion abilities, which were inhibited by LINC01420 knockdown, were reversed upon miR-320a inhibition and cell functions were instead promoted. (** *P* < 0.01, *** *P* < 0.001 compared to inhibitor NC; # *P* < 0.05, ## *P* < 0.01, ### *P* < 0.001 compared to si-LINC01420 + inhibitor NC)

miR-320a inhibitor significantly suppressed miR-320a expression compared with inhibitor NC, as shown in Fig. 5A (*P* < 0.001). It has been confirmed that LINC01420 knockdown promoted miR-320a expression in this study (*P* < 0.001). However, the results of co-transfected si-LINC01420 and miR-320a inhibitor indicated that miR-320a inhibitor suppressed the situation in which LINC01420 knockdown promoted miR-320a expression, and decreased the expression level of miR-320a (Fig. 5A, all *P* < 0.001).

In addition, the effects of co-transfecting si-LINC01420 and miR-320a inhibitor on cell proliferation, migration and invasion were investigated in this study. After transfection with miR-320a inhibitor were significantly enhanced the abilities of cell proliferation (*P* < 0.01), migration (*P* < 0.001), and invasion (*P* < 0.001) compared with inhibitor NC (Fig. 5B-D). Whereas si-LINC01420 and inhibitor NC inhibited the abilities of cell proliferation (*P* < 0.01), migration (*P* = 0.007) and invasion (*P* = 0.0004) compared with only inhibitor NC (Fig. 5B-D). However, in cells simultaneously silencing LINC01420 and miR-320a, it was found that cell proliferation (*P* < 0.05), migration (*P* < 0.001), and invasion (*P* < 0.001) abilities, which were inhibited by LINC01420 knockdown, were reversed upon miR-320a inhibition and cell functions were instead promoted (Fig. 5B-D).

### Target prediction and function analysis of miR-320a

**Fig. 6:**
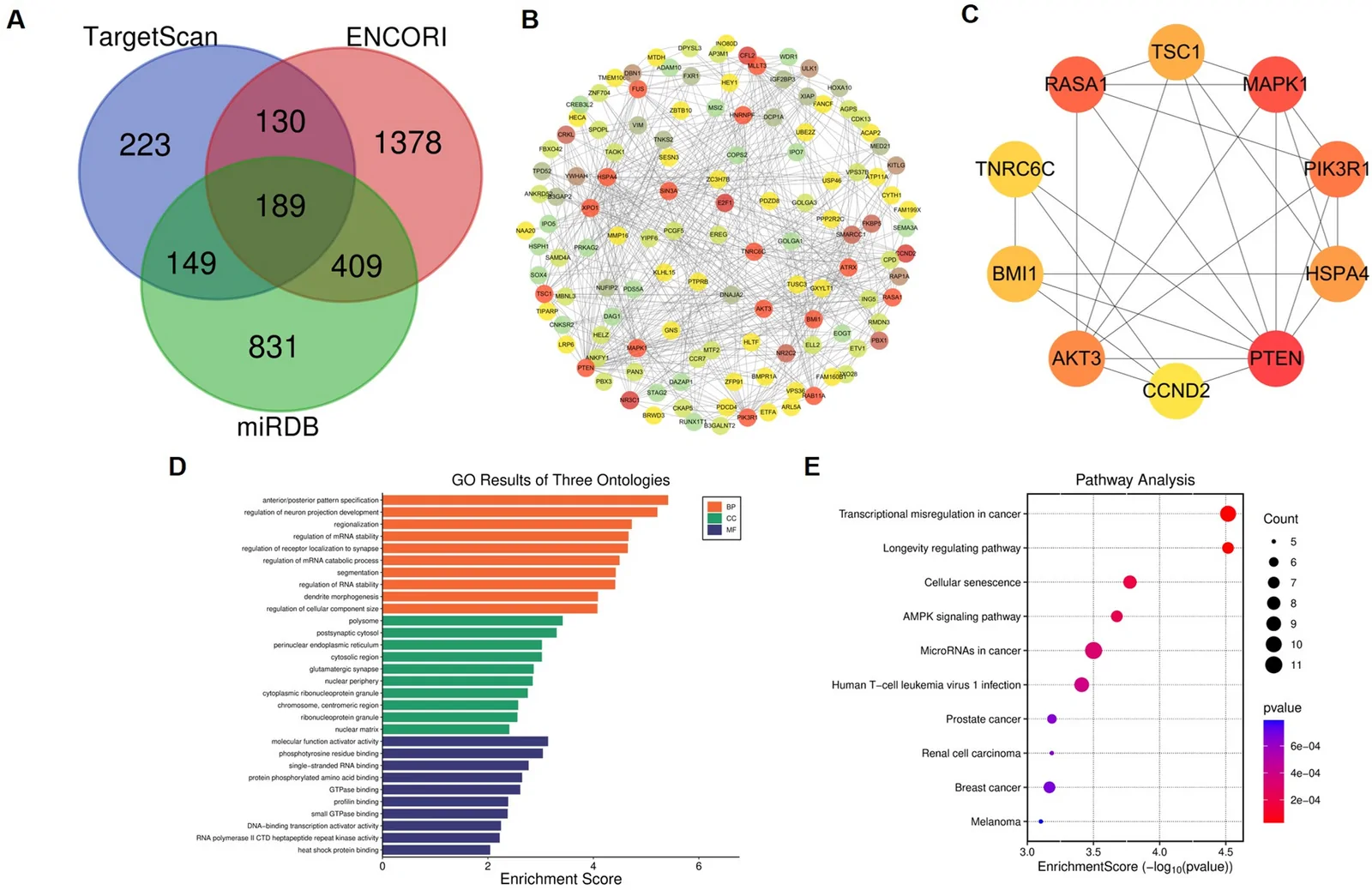
Target prediction and function analysis of miR-320a. **A**. A of 189 overlapping targets of miR-320a were identified in the TargetScan, ENCORI, and miRDB database. **B**. Analysis of potential target genes of miR-320a by PPI method. **C**. Screening of miR-320a key target genes. **D**. Bar graph of GO analysis of target genes of miR-320a. **E**. KEGG Pathway bubble map of target genes of miR-320a

The target genes of miR-320a were predicted by TargetScan, ENCORI, and miRDB databases. The Venn diagram results showed that there were 189 overlapping target genes **(**Fig. 6A**)**. Subsequently, these 189 target genes were imported into the STRING database to construct a PPI network **(**Fig. 6B**)**. Cytoscape was used to screen hub genes in the PPI network, with key genes including PTEN, MAPK1, RASA1, PIK3R1, AKT3, HSPA4, TSC1, BMI1, TNRC6C, and CCND2 identified **(**Fig. 6C**)**. These overlapping targets were further subjected to GO and KEGG analyses. As shown in Fig. 6D and Supplementary Table 1, in terms of GO molecular function (MF), these overlapping targets were mainly enriched in entries such as molecular function activator activity and single-stranded RNA binding; in biological process (BP), they were mainly enriched in entries such as regulation of mRNA stability and regulation of RNA stability; in cellular component (CC), they were mainly enriched in entries such as polysome. Subsequent KEGG pathway enrichment analysis showed that these overlapping genes were involved in pathways such as Transcriptional misregulation in cancer, AMPK signaling pathway and PI3K-Akt signaling pathway **(**Fig. 6E and Supplementary Table 2**)**.

## Discussion

Many studies have been substantiated that aberrant expression of lncRNAs was related to the progression of various cancers [5, 21]. Similar findings were also observed in CCA, such as lncRNA SNHG3 expression was abnormally up-regulated in CCA tissues and finally affecting proliferation and migration of CCA cells [22]. Upregulation of DLGAP1-AS2 level in CCA has been considered as a biomarker [23]. LINC00844 and LINC01123 have also been confirmed to serve as biomarkers for the prognosis of CCA [24–26]. In our current study, we discovered that the expression level of LINC01420 in the CCA tissue was substantially higher than that in the normal tissue using the information from the GEPIA database. Further validation of 96 clinical samples and cell lines showed that the expression levels of LINC01420 were significantly increased in CCA tumor tissues and cells compared with normal, and LINC01420 expression was significantly higher in advanced TNM stage than in early TNM stage. Moreover, analysis of clinicopathological features showed that LINC01420 expression was significantly correlated with tumor size, lymph node metastasis and TNM stage in CCA. Kaplan-Meier curves indicated that high LINC01420 was associated with worse overall survival prognosis in CCA tissues collected for this study. The Cox regression analysis further confirmed that LINC01420 is an independent influencing factor for the prognosis of patients with CCA. It is worth noting that the abnormal expression of LINC01420 has also been reported in other tumors. For instance, in nasopharyngeal carcinoma, LINC01420 is also highly expressed, and its high expression is significantly associated with a poor prognosis for the patients [15, 27]. On the basis of the above, we infer that LINC01420 may be involved in the progression of CCA.

There have been various researches indicated that lncRNAs could able to regulate the tumor biological functions of CCA [28, 29]. For example, lncRNA CCAT1 promoted migration and invasion of CCA via sponging miR-181a-5p to regulate ROCK2 [30]. Li et al. showed that knockdown of NEAT1 markedly restrained cell proliferation, invasion, and migration and stimulated cell apoptosis in CCA cells [31]. In order to investigate the biological function of LINC01420 in CCA, cell proliferation, migration and invasion assays were performed in this study. The results demonstrated that knockdown of LINC01420 inhibited CCA cell proliferation, migration and invasion. Moreover, it has been demonstrated that LINC01420 influenced NPC biological function [15]. Based on the classical mechanism by which lncRNAs regulates target miRNA through the ceRNA model [16, 17]. This study further explored the interaction and regulatory patterns between LINC01420 and miR-320a. The prediction results of LncBase Predicted v.2 showed that LINC01420 and miR-320a were complementary binding sequences. Moreover, the expression level of miR-320a was downregulated in CCA tumor tissues, and the expression levels of LINC01420 and miR-320a are negatively correlated. To clarify the ceRNA relationship between LINC01420 and miR-320a, we conducted cell experiments. After knocking down LINC01420, the proliferation, migration, and invasion abilities of CCA cells were all inhibited. However, when LINC01420 was knocked down simultaneously and miR-320a was inhibited, the aforementioned inhibitory effect was significantly reversed. This result confirmed that LINC01420 can act as a ceRNA to bind to miR-320a and participate in the functional regulation of CCA cells by regulating its expression.

To further investigate the underlying molecular mechanism by which miR-320a exerts its influence on CCA, we obtained 189 overlapping target genes through predictive analyses. Through PPI prediction and screening of these overlapping target genes, 10 hub genes including PTEN, MAPK1, RASA1, PIK3R1, AKT3, HSPA4, TSC1, BMI1, TNRC6C, and CCND2 were identified. Among them, PTEN has been extensively studied in CCA and is crucial for the regulation of cell proliferation, differentiation and apoptosis. Jiang et al. demonstrated that miR-181b-5p promotes cell growth, migration, and invasion by targeting PARK2 and modulating the PTEN/PI3K/AKT axis [32]. Additionally, Zhang et al. revealed that miR-22-3p regulates the proliferation and migration of cholangiocarcinoma cells by inhibiting 5-fluorouracil resistance through the PTEN/PI3K/AKT axis [33]. Based on these findings, we speculate that miR-320a may alleviate the progression of cholangiocarcinoma by directly targeting and binding to the PTEN/PI3K/AKT axis, thereby regulating cellular biological functions. KEGG pathway analysis revealed that these genes are significantly enriched in tumor-related signaling pathways, such as the Transcriptional misregulation in cancer signaling pathway, AMPK signaling pathway and MicroRNAs in cancer pathway (Supplementary Table S2). These pathways are known to play crucial roles in tumor initiation and progression. Combined with the results of GO functional annotation (Supplementary Table S1), it suggests that miR-320a might be involved in these pathways through the regulation of these hub genes, consequently affecting the biological behavior of CCA cells.

Although this study initially revealed the core mechanism by which LINC01420 acts as a ceRNA to regulate miR-320a and influence the progression of CCA, there are still some limitations. Firstly, this study lacks in-depth verification of the specific molecular mechanism by which the LINC01420/miR-320a signaling axis affects the functions of CCA cells through regulating specific target genes. In future studies, we will verify the role of the target genes in this signaling axis to further improve the research on the molecular mechanism. Secondly, we used two cell lines, HuCCT1 (hepatic intrahepatic cholangiocarcinoma cell line) and TFK-1 (hepatic extrahepatic cholangiocarcinoma cell line). These two cell lines alone cannot comprehensively cover the molecular heterogeneity within both intrahepatic and extrahepatic CCA. This may to some extent limit the general applicability of the research conclusions. In subsequent studies, we will expand the range of cell line selection, include more CCA cell lines with different differentiation degrees and different genetic backgrounds, and simultaneously conduct verification using primary cells from clinical sample sources to enhance the representativeness and reliability of the research results.

## Conclusion

In conclusion, LINC01420 expression was significantly increased in CCA tumor tissues and cells, high LINC01420 was associated with tumor size, lymph node metastasis, TNM stage and worse overall survival prognosis, and may serve as a prognostic biomarker for CCA patients. In addition, in CCA cells, knockdown of LINC01420 inhibited CCA cell proliferation, migration and invasion by acting as a ceRNA of miR-320a. All the findings of this study allow researchers to gain new understanding about CCA pathogenesis, and suggest that aberrant LINC01420 and miR-320a may serve as a novel biomarkers and therapeutic targets in CCA.

## Supplementary Information

Below is the link to the electronic supplementary material.

Supplementary Material 1

Supplementary Material 2

## Data Availability

The data used and analyzed can be obtained from the corresponding author under a reasonable request.
